# Relocation Post-*Dobbs* Among Clinicians Providing Abortions

**DOI:** 10.1001/jamanetworkopen.2025.14884

**Published:** 2025-06-11

**Authors:** Dana Howard, Marta Bornstein, Jocelyn Wascher, Alison H. Norris, Katherine Rivlin

**Affiliations:** 1 Division of Bioethics, Department of Biomedical Education and Anatomy, The Ohio State University College of Medicine, Columbus; 2 Department of Health Promotion, Education, and Behavior, Arnold School of Public Health, University of South Carolina, Columbia; 3 Department of Obstetrics and Gynecology, University of Chicago, Chicago, Illinois; 4 Division of Epidemiology, College of Public Health and Medicine, The Ohio State University, Columbus; 5 Department of Obstetrics and Gynecology, University of Chicago, Chicago, Illinois

## Abstract

This survey study compares the proportion of abortion-providing clinicians who changed primary state of practice after the *Dobbs v Jackson Women’s Health Organization *decision in 2022.

## Introduction

Intensifying state-level abortion restrictions following the *Dobbs v Jackson Women’s Health Organization* (2022) decision could lead clinicians to leave states that ban abortion.^1^ While large-scale changes are not yet apparent among obstetrician-gynecologists, the abortion care workforce may be uniquely at risk.^2^ We examined the proportion of abortion-providing clinicians who changed primary state of practice, comparing those who left states that banned abortion with those who left states that did not ban abortion after *Dobbs*, and the ban status of the states to which they relocated.

## Methods

This survey study was approved by The Ohio State University institutional review board. From May to December 2023, we disseminated an electronic, purposive survey to national professional listservs for abortion-providing clinicians and through snowball sampling.^3^ Following AAPOR reporting guidelines, we recruited clinicians (physicians, advanced practice clinicians, and nurses) who provided abortion the year before *Dobbs* and/or at the time of the survey.^4^ Surveys queried primary practice state in the year before *Dobbs* and at the time of the survey. Electronic informed consent was obtained prior to the survey.

We categorized states based on abortion policy. Any state with a near-total abortion ban or 6-week gestational abortion ban in effect at any time following *Dobbs* (June 2022) and our survey’s close (December 2023) was categorized as a ban state. All other states were categorized as no-ban. If clinicians reported a different primary practice state in the year before *Dobbs* compared with their primary practice state during the survey, they were categorized as relocating primary practice state.

Finally, we assessed the proportion of clinicians who changed primary practice state from a ban state to a no-ban state. Analyses were conducted in Stata version 18 (StataCorp) in November 2024. The threshold for statistical significance was a 2-sided *P* < .05.

## Results

Of 388 respondents, 346 (89%) met eligibility and consented. We excluded respondents not practicing before *Dobbs* (19 respondents) or not reporting primary practice state before and after *Dobbs,* for an analytic sample of 305 (79%). Most respondents (277 respondents [91%]) reported providing some nonabortion health care (Table 1). Respondents reported primarily practicing in 44 states and Washington DC, with 227 respondents (74%) practicing in no-ban states and 78 (26%) practicing in ban states before *Dobbs* (Table 1).

**Table 1.** Characteristics of Clinicians Providing Abortion Before *Dobbs v Jackson Women’s Health Organization *

Characteristic	Clinicians, No. (%) (N = 305)
State of primary practice^a^	
No-ban state	227 (74)
Ban state	78 (26)
Region of primary practice	
West	61 (20)
Southwest	29 (10)
Midwest	63 (21)
Southeast	77 (25)
Northeast	75 (25)
Clinical care	
Provided some nonabortion care	277 (91)
Only provided abortion care	28 (9)
Healthcare role	
Obstetrics and gynecology	166 (54)
Family medicine	55 (18)
Other physician	5 (2)
Advanced practice clinician, nurse practitioner	41 (13)
Advanced practice clinician, physician assistant	6 (2)
Advanced practice clinician, certified nurse midwife	12 (4)
Advanced practice clinician, other	2 (1)
Nurse	18 (6)
Complex Family Planning Fellowship (physicians only), No./total No. (%)	
Yes (completed or currently completing)	120/226 (53)
No	106/226 (47)
In training	
Yes	26 (9)
No	279 (91)
Primary practice setting	
Community hospital	12 (4)
Academic hospital	85 (28)
State-funded institution	4 (1)
Planned Parenthood	115 (38)
Independent clinic	73 (24)
Telehealth	8 (3)
Other	6 (2)
Missing	2 (1)

^a^ Ban or 6-week ban was coded based on the implementation of a near total ban or a 6-week gestational ban on abortion between June 2022 (*Dobbs*) and December 2023 (the end of our survey). These states include Alabama, Arizona, Arkansas, Georgia, Idaho, Indiana, Iowa, Kentucky, Louisiana, Mississippi, Missouri, North Dakota, Ohio, Oklahoma, South Carolina, South Dakota, Tennessee, Texas, West Virginia, and Wisconsin.

Overall, 47 respondents (16%) relocated primary practice state. Clinicians practicing before *Dobbs* in states that would ban abortion were much more likely to relocate primary practice state than those in no-ban states (27 respondents [42%] vs 20 respondents [9%]; *P* < .001) (Table 2).

**Table 2.** Changes in Practice After *Dobbs v Jackson Women’s Health Organization *

Characteristic	Clinicians by pre-*Dobbs* primary state of practice, No./total No. (%)			*P* value
	Total	No ban	Ban or 6-wk ban^a^	
Abortion provision				
Not currently providing	17/305 (6)	4/227 (2)	13/78 (17)	<.001
Currently providing (post-*Dobbs*)	288/305 (94)	223/227 (98)	65/78 (83)	
Moved primary practice state				
Did not move	241/288 (84)	203/227 (91)	38/78 (58)	<.001
Moved	47/288 (16)	20/227 (9)	27/78 (42)	
Move type^a^				
Ban to ban	3/47 (6)	NA	3/27 (11)	NA
No-ban to ban	2/47 (4)	2/20 (10)	NA	
Ban to no-ban	24/47 (51)	NA	24/27 (89)	
No-ban to no-ban	18/47 (38)	18/20 (90)	NA	

^a^ Ban or 6-week ban was coded based on the implementation of a near total ban or a 6-week gestational ban on abortion between June 2022 (*Dobbs*) and December 2023 (the end of our survey). These states include Alabama, Arizona, Arkansas, Georgia, Idaho, Indiana, Iowa, Kentucky, Louisiana, Mississippi, Missouri, North Dakota, Ohio, Oklahoma, South Carolina, South Dakota, Tennessee, Texas, West Virginia, and Wisconsin.

Of those who relocated from ban states, most (24 of 27 respondents [89%]) relocated to no-ban states. Among those who relocated from no-ban states, most relocated to another no-ban state (18 of 20 respondents [90%]) (Table 2). Some respondents (17 of 305 respondents [6%]) stopped providing any abortion care after *Dobbs,* mostly in ban states (13 respondents total) (Table 2).

## Discussion

This survey study found that after *Dobbs,* 42% of survey respondents who provided abortions in states banning abortion relocated to another state. Almost all clinicians who relocated from any policy context relocated to states not banning abortion. We document practice relocation rates vastly exceeding those of obstetrician-gynecologists from 2005 to 2015, and among obstetrician-gynecologists post-*Dobbs*.^2,5^ Broader reproductive health care workforce patterns can take years to develop. Our findings among clinicians providing abortion give early insights into future workforce shifts. Given that most study respondents provided both abortion and nonabortion health care, these accelerated relocations have implications for abortion access and for the broader maternal health workforce, exacerbating health care deserts and outcome disparities.^6^

This study has limitations. Our sample is purposive because listservs used for recruitment protect member identities and numbers and our study may have selection bias. These privacy measures are necessary for study participant protection given the sensitive nature of abortion provision.^4^ Our study reflects abortion-providing clinicians, not the broader reproductive and maternal health care workforce.

When clinicians cannot provide the standard of care, they may leave. Patients left behind could lack access to reproductive and maternal health care.

## Notes

Supplement.
Data Sharing Statement
