# Supplementary material for: Relocation Post-Dobbs Among Clinicians Providing Abortions
Source: JAMA Netw Open. 2025 Jun 11;8(6):e2514884. doi: 10.1001/jamanetworkopen.2025.14884 (PMC12159771; doi:10.1001/jamanetworkopen.2025.14884)
Supplement: Supplement. — Data Sharing Statement [file jamanetwopen-e2514884-s001.pdf]

Data Sharing Statement

Howard. Relocation Post-Dobbs Among Clinicians Providing Abortions. *JAMA Netw Open*. Published June 11, 2025. doi:10.1001/jamanetworkopen.2025.14884

Data
Data available: No

Additional Information
Explanation for why data not available: Due to the sensitive nature of abortion provision, we are not making data available regarding individual responses. These responses, while de-identified, may include sensitive information.
